# The impact of maternal eating disorders on breastfeeding practices: a systematic review

**DOI:** 10.1007/s00737-021-01103-w

**Published:** 2021-04-08

**Authors:** Anna Kaß, Annica Franziska Dörsam, Magdalene Weiß, Stephan Zipfel, Katrin Elisabeth Giel

**Affiliations:** 1 Department of Psychosomatic Medicine and Psychotherapy, University Hospital Tübingen, 72076 Tübingen, Germany; 2 Competence Center for Eating Disorders (KOMET), 72076 Tübingen, Germany; 3 Department of Obstetrics and Gynecology, University Hospital, 72076 Tübingen, Germany

**Keywords:** Breastfeeding, Lactation, Eating disorders, Anorexia nervosa, Bulimia nervosa, Pregnancy

## Abstract

Breastfeeding is an effective way to protect and promote the health of the infant and mother. Cultural, social, economic, medical, or psychological factors might interfere with successful breastfeeding. Therefore, maternal eating disorders (EDs) may have detrimental effects on the decision of breastfeeding initiation and on its continuation. There is limited knowledge about the breastfeeding practices of mothers with EDs. We performed a systematic review to generate more evidence in this area. A search was conducted in PubMed and PsycINFO, and several journals were hand searched for relevant publications. Of *N* = 3904 hits, 13 full texts were included in the qualitative analysis. The findings on total duration of BF between mothers with and without EDs were mixed, but women with EDs showed more negative experiences and emotional problems during BF. There was not enough evidence to conclude on breastfeeding initiation, or on the duration of exclusive breastfeeding. Maternal EDs might have a negative impact on BF practices with possible negative effects on the maternal-child feeding environment. Further studies with comparable data and information on the women’s partners’ attitudes about breastfeeding are needed.

## Introduction

According to current scientific evidence, breastfeeding is an effective way to protect and promote infant health (Koletzko et al. 2013; Westerfield et al. 2018). It is recommended to breastfeed exclusively for at least 4 months, ideally for 6 months, and additionally for up to 2 years (WHO 2003). It is not only the optimal way of feeding the baby but also strengthens the emotional bond between mother and child and can prevent allergies and other diseases in both mother and child (Koletzko et al. 2010). Breastfed children show a decreased risk of infections, diabetes, and overweight in later life (Victora et al. 2016). For mothers, breastfeeding (BF) offers protection against breast cancer and might even protect against ovarian cancer and type 2 diabetes (Victora et al. 2016), besides the involution and regeneration of the body (Nurjanna et al. 2019). However, some women face barriers to BF, which may include delivery via cesarean section, low socioeconomic status, lack of social support, the need to return to work, and poor breastfeeding education (Sayres and Visentin 2018), as well as cultural beliefs (Hannula et al. 2008). Additionally, mothers suffering from medical and/or psychological disease, such as obesity (Lepe et al. 2011) or depression (Dias and Figueiredo 2015), might experience difficulties with BF.

The quality and amount of the mother’s milk are dependent on her nutrition and lifestyle (Koletzko et al. 2013). Studies show that nutritional deficiencies result in lower concentrations of nutrients in breast milk (Daniels et al. 2019; Machado et al. 2019). For instance, low vitamin A, vitamin E (Machado et al. 2019), niacin, and riboflavin intakes (Daniels et al. 2019) are reflected in the milk’s composition. To provide all necessary macro- and micronutrients for the child, the mother needs to maintain a diverse, well-balanced, and regular diet (Koletzko et al. 2013; Prell and Koletzko 2016).

Since dysfunctional eating behaviors are the main characteristics of eating disorders (EDs) (APA 2013), mothers suffering from EDs may have difficulties meeting the nutritional recommendations during BF. An altered micronutrient status, most commonly vitamin A deficiencies, was observed in patients with anorexia nervosa (Achamrah et al. 2017). Current research shows that approximately 1.5 to 7.6% of pregnant women are affected by EDs (Bye et al. 2020). Although ED symptoms often decrease during pregnancy, they return after childbirth in many cases (Knoph et al. 2013; Watson et al. 2013), which is the time frame for BF initiation.

Anorexia nervosa (AN), bulimia nervosa (BN), and binge eating disorder (BED) and “other specified feeding or eating disorder” (OSFED) represent the specific eating disorders (EDs) delineated in the Diagnostic and Statistical Manual of Mental Disorders, 5th Edition (DSM-V) (APA 2013). All forms of EDs are characterized—at varying degrees—by dietary restraint, binge eating, and distorted body-related attitudes (Treasure et al. 2010; Treasure et al. 2020). Briefly, the central feature of AN is extremely low body weight and a fear of gaining weight (Resmark et al. 2019); BN comprises repeated binge eating, followed by inappropriate compensatory behaviors to prevent weight gain (e.g., self-induced vomiting) (Falkai et al. 2018; Treasure et al. 2010; Treasure et al. 2020). BED is also associated with recurring binge eating episodes, accompanied by feelings of lack of control, guilt, embarrassment, or disgust (APA 2013; Falkai et al. 2018). The diagnosis of OSFED includes all forms of disordered eating, which are not fully covered by the diagnostic criteria of the other ED subtypes (APA 2013).

There is evidence that mothers with EDs show altered attitudes toward child feeding. For example, women with BN might experience a fear to binge eat the baby’s food (Fahy and Treasure 1989; Stapleton et al. 2009). This may lead to extended duration of BF in mothers with BN to avoid the handling of solid foods. On the other hand, restrictive energy intake of mothers with AN might result in insufficient milk production (Waugh and Bulik 1999). Therefore, cessation of BF in AN patients might be earlier than intended (Waugh and Bulik 1999). Women with EDs reported that they could either not start BF due to the strong need to lose weight or to maintain restrictive eating patterns (Stapleton et al. 2008). On the other hand, women reported that BF meant burning calories, which was the reason for them to start BF (Stapleton et al. 2008). Previous evidence on BF practices in women with EDs therefore expresses an ambivalence of these mothers between ED-related concerns and behaviors on the one hand and the wish of satisfying the baby’s needs on the other hand. This can result in feelings of guilt and shame in many mothers with EDs (Stapleton et al. 2008). A mechanism which might contribute to these BF difficulties in the ED spectrum is oxytocin functioning. The hypothalamic neuropeptide oxytocin regulates a broad range of social behavior, is involved in attachment, e.g., within mother-child interactions, and is released during BF (Marlin and Froemke 2017). Recently, it has been outlined that oxytocin also contributes to the regulation of eating behavior and might play a role in the disease process of EDs (Giel et al. 2018). Although the specific interplay between behavioral expressions and hormonal regulation is not yet clear, oxytocin functioning might underlie some BF difficulties seen in women with EDs and might even contribute to an intergenerational transmission of EDs via attachment experiences in early childhood (Giel et al. 2018).

As there are various contrasting results regarding BF practices of mothers with lifetime EDs, the present systematic review is intended to provide more detailed evidence regarding overall BF behavior of mothers with current or active EDs, including BF duration as well as physical and emotional barriers to BF. Previously published reviews on this topic had different primary aims (Behar and Arancibia 2014; Martini et al. 2020). Therefore, the main research questions of the present systematic review are:

- Is there a difference in duration of exclusive or partial BF after childbirth in women with a history of EDs compared to healthy women?
- Is there a difference regarding physical and emotional problems during BF in women with a history of EDs compared to healthy women?

## Materials and methods

The present review was conducted using scientific papers examining the influence of maternal EDs on BF behavior. The research strategy and data extraction process were based on the guidelines of the Preferred Reporting Items for Systematic Reviews and Meta-Analyses (PRISMA) (Moher et al. 2009).

### Terminology

Exclusive BF is defined as “breastfeeding without any supplements of formula milk or solid food” (Kersting et al. 2020; Torgersen et al. 2010). In many studies, partial BF, defined as “continued breastfeeding, with possible supplementation of milk or solids,” is a more common mode of BF (Kersting et al. 2020; Torgersen et al. 2010), because exact information on supplementation and formula is sometimes missing.

### Research strategy and data extraction

The systematic search was conducted until September 2020 (see Table 1 for detailed information on research strategy and data extraction process). The full search path for PubMed is displayed in the supplement section.

**Table 1:**
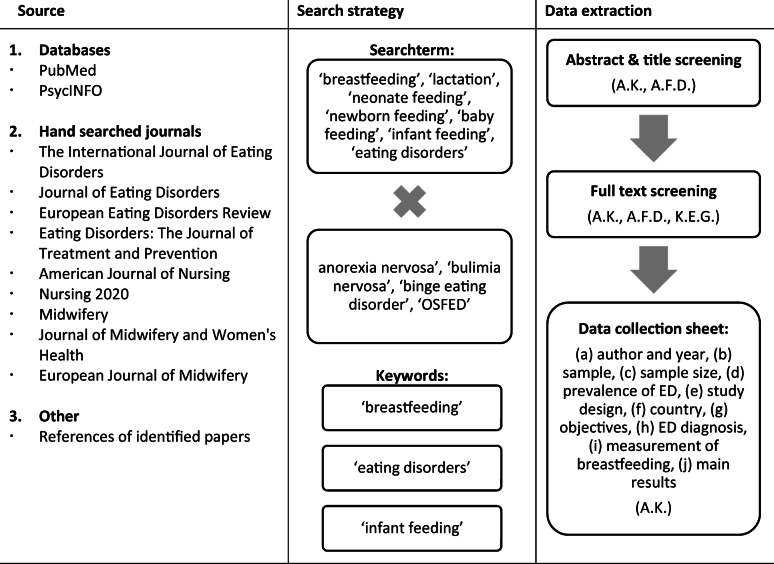
Overview of the research strategy and data extraction process

Note: The initials of authors who were integrated in the respective processes are displayed in brackets

### Eligibility criteria

To qualify for this review, papers had to meet criteria defined according to the five PICOS dimensions: participants (P), interventions (I), comparators (C), outcomes (O), and study design (S) (Moher et al. 2009). Due to the fact that BF is a natural behavior and that the actual behavior of mothers was of interest for this review, the PICOS scheme was slightly modified by replacing the item “interventions” with “investigations.”

#### Participants

Participants included mother-child dyads, whereby the mother had an active or past ED. EDs had to be diagnosed according to DSM criteria and included the diagnoses of AN, BN, BED, and OSFED.

#### Investigations

Studies investigating the duration of BF and/or the occurrence of physical or psychological problems during BF in women with active or past EDs were included in this review. Reported problems should be based on medical records, but also self-reported perceptions were considered. Additionally, feelings and general attitudes toward BF were relevant for the present review. This review also included studies reporting on scheduled feeding, as well as differences in the feeding behavior of mothers with EDs between daughters and sons.

#### Comparators

Control groups included healthy mother-child dyads with mothers who did not suffer from any lifetime EDs. Studies were excluded if a control group was missing.

#### Outcome measures

The primary outcome measure for this review was BF behavior in mothers with EDs including total BF duration, BF initiation, and problems associated with BF. Secondary outcome measures were feelings during BF, scheduled feeding, and general attitudes toward child feeding.

#### Study design

Cross-sectional and longitudinal studies as well as case-control and cohort studies investigating BF behavior of mothers with active or past EDs were included. No restriction regarding the publication date was imposed. Unpublished material (www.opengrey.eu) was also included. Secondary data analyses such as reviews and book chapters as well as case reports, abstracts, and dissertations were excluded. Publication languages included English, German, French, and Spanish.

### Quality assessment of included studies

The first and the second authors rated the studies according to quality aspects of the Newcastle-Ottawa Scale (NOS) (Wells et al. 2008) for quality assessment of cohort and case control studies (Table 3). The quality of the studies was assessed by the scores achieved in the categories (1) selection of the cohort group or cases, (2) comparability with controls, and (3) outcome (cohort studies)/exposure (case control studies). The maximum score achievable was nine. Points were given for example for representativeness of the study group, a community selection of the control group, the ascertainment of exposure by secure records, and independent blind assessment of the outcome.

## Results

**Fig. 1:**
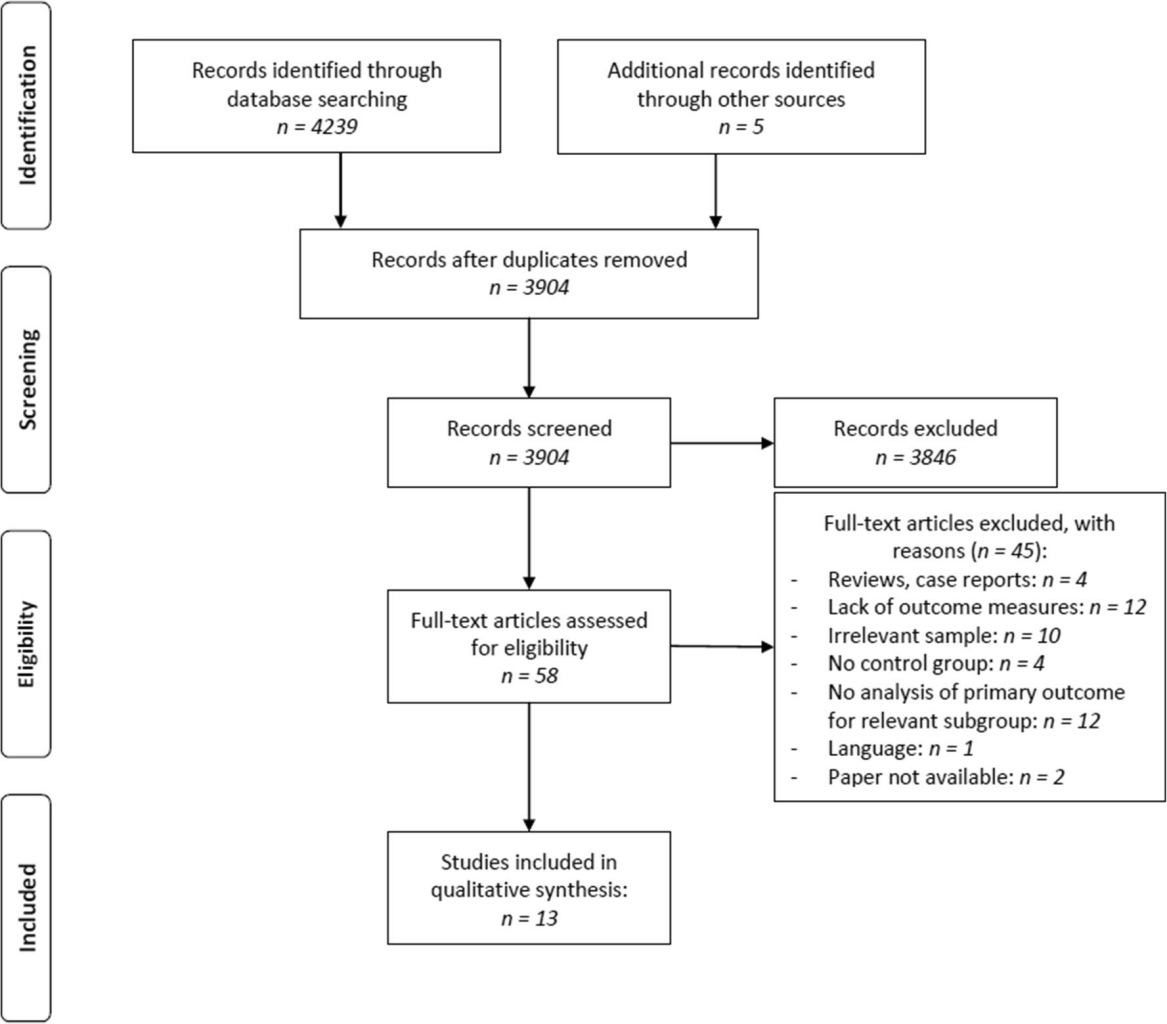
Systematic review search process displayed in the PRISMA flow diagram

In total, 13 publications on 12 different studies qualified for inclusion in this review. Publication dates ranged from 1988 (Brinch et al. 1988; *n* = 50) to 2019 (Martini et al. 2019; *n* = 99). The flow diagram visualizes the process of the study selection (Fig. 1).

The sample size in the included studies ranged from *n* = 20 to *n* = 53,879. The combined samples of all studies included *n* = 78,259 women, of which *n* = 4667 had been diagnosed with a lifetime ED. A total of *n* = 73,592 women without any history of EDs were included as a control group. Since the study by Torgersen et al. published in 2015 (Torgersen et al. 2015; *n* = 53,879) was the follow-up investigation of the paper published in 2010 (Torgersen et al. 2010; *n* = 39,355), this sample was only considered once in this review. Four participants were diagnosed with co-occurring AN and BN (Allen et al. 2014; *n* = 166; Waugh and Bulik 1999; *n* = 20), which were re-classified as OSFED. Information on the distribution of ED diagnoses can be found in Table 2.

**Table 2:** Distribution of ED diagnoses in the overall sample

	ED					Control
	AN	BN	BED	OSFED	Unspecified ED diagnosis	
*N* subtypes	436	655	2502	62	1012	73,592
*N* total	4667					

Abbreviations: *N* total number, *ED* eating disorder, *AN* anorexia nervosa, *BN* bulimia nervosa, *BED* binge eating disorder, *OSFED* other specified feeding and eating disorders

The investigations took place at very different times in the lives of the women. Some publications assessed the age of the women at their first delivery; others assessed the current age at a follow-up. The body mass index (BMI) was reported as either the lowest lifetime BMI, the postpartum BMI, or the BMI years after the last delivery. Therefore, we refrain from specifying the average age or BMI of the participants, as there is no comparability.

The selection contains six longitudinal studies, of which three were part of large population-based studies (Agras et al. 1999; *n* = 194; Martini et al. 2019; *n* = 99; Micali et al. 2009; *n* = 12,050; Nguyen et al. 2017; *n* = 6196; Torgersen et al. 2010; *n* = 39,355; Torgersen et al. 2015; *n* = 53,879; Waugh and Bulik 1999; *n* = 20). In addition, there was one cross-sectional study (Larsson and Andersson-Ellström 2003; *n* = 454) and five cohort studies (Allen et al. 2014; *n* = 194; Brinch et al. 1988; *n* = 50; Evans and Le Grange 1995; *n* = 20; Hoffman et al. 2014; *n* = 50; Popovic et al. 2018; *n* = 5081). In all cases, mothers with lifetime EDs were compared with control women without any history of EDs.

Maternal EDs were assessed with self-report questionnaires like the Eating Attitude Test (EAT) or the Eating Disorder Examination Questionnaire (EDE-Q) in six of the included studies (Evans and Le Grange 1995; *n* = 20; Larsson and Andersson-Ellström 2003; *n* = 454; Micali et al. 2009; *n* = 12,050; Nguyen et al. 2017; *n* = 6196; Popovic et al. 2018; *n* = 5081; Torgersen et al. 2010; *n* = 39,355; Torgersen et al. 2015; *n* = 53,879). The other studies combined self-report questionnaires with a clinical/psychiatric interview such as the Structured Clinical Interview for DSM-III/IV (SCID-I), the EDE Interview, the Diagnostic Interview for Genetic Studies, or other unspecified interviews (Agras et al. 1999; *n* = 194; Allen et al. 2014; *n* = 166; Hoffman et al. 2014; *n* = 50; Martini et al. 2019; *n* = 99; Waugh and Bulik 1999; *n* = 20). In one case, the ED diagnosis was based on the interview alone (Waugh and Bulik 1999; *n* = 20). In one study, participants were recruited from the total of patients of a specialized ED clinic; therefore, the diagnosis had been made in the past (Brinch et al. 1988; *n* = 50). In eight of 12 cases, the diagnosis was made based on DSM-III, DSM-IV, or DSM-V criteria (Agras et al. 1999; *n* = 194; Allen et al. 2014; *n* = 166; Evans and Le Grange 1995; *n* = 20; Hoffman et al. 2014; *n* = 50; Martini et al. 2019; *n* = 99; Nguyen et al. 2017; *n* = 6196; Torgersen et al. 2010; *n* = 39,355; Torgersen et al. 2015; *n* = 53,879; Waugh and Bulik 1999; *n* = 20).

### Quality assessment

The majority of studies showed a solid study design and convincing results and were rated as strong (Agras et al. 1999; *n* = 194; Allen et al. 2014; *n* = 166; Evans and Le Grange 1995; n = 20; Hoffman et al. 2014; *n* = 50; Martini et al. 2019; *n* = 99; Micali et al. 2009; *n* = 12,050; Waugh and Bulik 1999; *n* = 20). In the remaining studies, points were deducted for (1) poor representativeness of cases and poor selection of controls (Brinch et al. 1988; *n* = 50) and (2) assessment of exposure based on self-report without secure record confirmation (Larsson and Andersson-Ellström 2003; *n* = 454; Nguyen et al. 2017; *n* = 6196; Popovic et al. 2018; *n* = 5081; Torgersen et al. 2010; *n* = 39,355; Torgersen et al. 2015; *n* = 53,879).

### Studies investigating breastfeeding initiation and total duration of breastfeeding

All 13 publications included investigations on BF initiation and/or total duration of BF. Information on feeding styles was collected with the help of self-report questionnaires such as the Infant Feeding Questionnaire (Martini et al. 2019; *n* = 99 ), a modified version of the Toddler Diet Questionnaire (Hoffman et al. 2014; *n* = 50), or questionnaires not further defined (Agras et al. 1999; *n* = 194; Larsson and Andersson-Ellström 2003; *n* = 454; Micali et al. 2009; *n* = 12,050; Nguyen et al. 2017; *n* = 6196; Popovic et al. 2018; *n* = 5081; Torgersen et al. 2010; *n* = 39,355; Torgersen et al. 2015; *n* = 53,879). Undefined questionnaires also included questions about the time point of BF termination, introduction to solid food, or use of formula. With this information, it was possible to draw a conclusion on exclusive BF duration. In the other cases, interviews were used to gather information on initial and total duration of BF (Allen et al. 2014; *n* = 166; Brinch et al. 1988; *n* = 50; Evans and Le Grange 1995; *n* = 20; Waugh and Bulik 1999; n = 20).

#### Studies showing no differences in breastfeeding duration in mothers with and without EDs

Seven studies did not show significant differences in the total BF duration in mothers with or without EDs, who initially breastfed their children (Agras et al. 1999; *n* = 194; Allen et al. 2014; n = 166; Brinch et al. 1988; *n* = 50; Evans and Le Grange 1995; n = 20; Hoffman et al. 2014; n = 50; Martini et al. 2019; *n* = 99; Nguyen et al. 2017; *n* = 6196). These studies included larger cohort studies as well as small sample sizes and participants with different kinds of ED diagnoses (AN, BN, OSFED, no specific ED, current ED, past ED). Total BF durations of mothers with EDs reached from 3 (Brinch et al. 1988; *n* = 50) to 12 months (Hoffman et al. 2014; *n* = 50).

Over 80% of women with EDs and 90% of the control women initially breastfed their child, without significant differences between groups (Evans and Le Grange 1995; *n* = 20). However, there was a tendency for mothers with a history of EDs (*n* = 591) to initiate BF less frequently (Nguyen et al. 2017; *n* = 6196).

Compared to controls, there were no significant differences in the percentage of mothers with ED who breastfed for more than 1 month or in the child’s age at solid food introduction, although a trend toward more children of mothers with EDs being introduced to solids after 7 months was indicated (*p* = 0.19) (Hoffman et al. 2014; *n* = 50). Contrary results were shown by Allen et al. (2014; *n* = 166), reporting women with EDs introduced their children to solid foods significantly earlier (*n* = 18; 15.96 weeks) compared to controls (*n* = 148; 21.86 weeks).

#### Studies showing shorter duration of breastfeeding in mothers with EDs

In total, four studies found a significantly shorter BF duration in mothers with EDs compared to mothers without EDs (Larsson and Andersson-Ellström 2003; *n* = 454; Popovic et al. 2018; *n* = 5081; Torgersen et al. 2010; *n* = 39,355; Torgersen et al. 2015; *n* = 53,879; Waugh and Bulik 1999; *n* = 20). The studies included a large prospective population-based cohort study (Torgersen et al. 2010; *n* = 39,355; Torgersen et al. 2015; *n* = 53,879) as well as studies with medium-sized samples with a spectrum of ED diagnoses (AN, BN, BED, OSFED).

With 80 to 100%, the vast majority of participants initiated BF after birth, without any significant differences between the mothers with and without EDs (Torgersen et al. 2010; *n* = 39,355; Torgersen et al. 2015; *n* = 53,879; Waugh and Bulik 1999; *n* = 20). Compared to controls, significantly less women with EDs exclusively breastfed their 3-month-old babies (73% ED vs. 84% control) (Larsson and Andersson-Ellström 2003; *n* = 454). Moreover, significantly more mothers with EDs (19%) did not breastfeed at all at 3 months postpartum compared to controls (7%) (Larsson and Andersson-Ellström 2003; *n* = 454). Waugh and Bulik (1999) found that mothers with EDs had significantly more difficulties in maintaining BF. Only 60% of the mothers with EDs continued BF until weaning compared to 100% of the controls (Waugh and Bulik 1999; *n* = 20).

There was also a significantly increased risk of cessation of BF before 6 months for the ED group compared to women without EDs (Popovic et al. 2018; *n* = 5081; Torgersen et al. 2010; *n* = 39,355; Torgersen et al. 2015; *n* = 53,879).

#### Studies showing longer duration of breastfeeding in mothers with EDs

Micali et al. (2009) used questionnaires at 1, 6, and 15 months postpartum on BF behavior, and the results included that women with EDs were significantly more likely to initially start BF (83% ED vs. 76% HC) and significantly less likely to stop BF in the child’s first year of life compared to controls. Mothers with BN were significantly most likely to continue BF beyond the first year of life (Micali et al. 2009; *n* = 12,050).

### Studies investigating emotions during breastfeeding and general attitudes toward breastfeeding

Most evidence on emotions during BF in mothers with EDs stems from small samples (*n* = 20) and older studies. Therefore, the results should be interpreted with caution. We identified three publications which assessed the emotions and attitudes of women with EDs during and toward BF (Evans and Le Grange 1995; *n* = 20; Larsson and Andersson-Ellström 2003; *n* = 454; Waugh and Bulik 1999; *n* = 20).

Women described feeling unsuccessful and guilty when their child would not eat (Evans and Le Grange 1995; *n* = 20). Sometimes they felt “waves of sadness” during BF (Evans and Le Grange 1995; *n* = 20) or showed strong embarrassment (Waugh and Bulik 1999; *n* = 20). Scheduled feeding led to anxiety and confusion in mothers with AN or BN when the child was hungry outside the recommended feeding times (Evans and Le Grange 1995; *n* = 20). There was also a nonsignificant tendency toward more negative experiences regarding breast changes, and fewer mothers with EDs reported a positive experience of BF compared to controls (Larsson & Andersson-Ellström) (*p* value not specified, 2003; *n* = 454).

### Studies investigating problems associated with breastfeeding

Concerning problems with BF, we identified five relevant studies (Agras et al. 1999; *n* = 194; Brinch et al. 1988; *n* = 50; Evans and Le Grange 1995; *n* = 20; Micali et al. 2009; *n* = 12,050; Waugh and Bulik 1999; *n* = 20). Problems with BF included difficulties during BF or problems which prevented BF initiation or forced the women to cease BF earlier than planned. The overall most common problem with BF in mothers with EDs, which all but one (Agras et al. 1999; *n* = 194) of the studies identified, was insufficient amounts of breast milk (Brinch et al. 1988; *n* = 50 ; Evans and Le Grange 1995; *n* = 20 ; Micali et al. 2009; *n* = 12,050 ; Waugh and Bulik 1999; *n* = 20). In almost all of these studies, the controls showed lower rates of problems with BF (Agras et al. 1999; *n* = 194; Evans and Le Grange 1995; *n* = 20; Micali et al. 2009; *n* = 12,050; Waugh and Bulik 1999; *n* = 20). The information on problems during BF was mostly based on mothers’ self-reports. In some cases, medical records were available, but it was not further defined if they included information on milk amount and other variables of BF (Agras et al. 1999; Brinch et al. 1988; Waugh and Bulik 1999).

Micali et al. (2009) showed similar rates of fast drinking in the children of mothers with AN (*n* = 234; 82.9%) and BN (*n* = 182; 81.9%) compared to controls (*n* = 9792; 85.4%) (Micali et al. 2009; *n* = 12,050), whereas daughters of mothers with EDs (*n* = 14) sucked significantly more rapidly compared to sons of mothers with EDs (*n* = 28) and compared to children of controls (*n* = 153) (Agras et al. 1999; *n* = 194). Daughters of mothers with EDs also showed significantly more vomiting during feeding compared to sons (Agras et al. 1999; *n* = 194). Children of mothers with EDs were reported to dawdle significantly more during feeding (62.5%) compared to controls (53.9%), but feeding durations were not different (Agras et al. 1999; *n* = 194).

Some children of mothers with BN had significantly higher refusal to eat solid food, which led to significantly longer duration of BF (Micali et al. 2009; *n* = 12,050). Mothers with AN especially showed significantly more early onset persistent feeding difficulties compared to controls or mothers with other EDs (Micali et al. 2009; *n* = 12,050). Problems included insufficient milk amounts, no milk, or mastitis, which pressured to early weaning (Brinch et al. 1988; *n* = 50) or to slow feeding, small quantitative feeding, and unsatisfied, still hungry infants after feeding (Micali et al. 2009; *n* = 12,050). One more reason for early cessation of BF was an allergy to breast milk (Evans and Le Grange 1995; *n* = 20).

### Studies investigating scheduled feeding

We identified three studies, which collected data on scheduled feeding concerning mothers with EDs (Agras et al. 1999; *n* = 194; Evans and Le Grange 1995; *n* = 20; Martini et al. 2019; *n* = 99). Schedules were based on recommendations by the mothers’ physicians or clinics (Evans and Le Grange 1995; *n* = 20). In the retrospective investigation by Evans and Le Grange (1995; *n* = 20), 55.6% of the children in the ED group were fed in a scheduled way compared to 10% in the control group, which was a significant difference. Martini et al. (2019) did not find a significant difference in scheduled feeding between mothers with current EDs, past EDs, and controls, although the implementation of scheduled feeding was slightly higher for mothers with current EDs at 6 months postpartum (Martini et al. 2019; *n* = 99). In contrast to these findings, mothers with EDs in the third study fed on a significantly less regular schedule (Agras et al. 1999; *n* = 194 ) (Table 3).

**Table 3:** Study characteristics

Author, Year	Sample	Sample size	Prevalence of ED	Study design	Country	Objectives	ED diagnosis	Measurement of BF	Main results	NOS rating
Agras et al. 1999	Mother-child dyads with or without history of EDs	*n* = 41 ED (active ED = 21) *n* = 153 NED	21% (partial) ED (AN = 2, BN = 17, BED = 22)	Longitudinal study	USA	Effect of maternal ED on their children regarding BF	Self-report questionnaire after delivery (EDI + TFEQ) + clinical interview	Self-report questionnaire (IFR) on 3 days/month until child was weaned: method of feeding, date of introduction to solid food	Compared to controls*:* *•* No difference in duration of BF • Daughters sucked more rapidly^*^, more dawdling and vomiting • *Women with ED fed on a less regular schedule • ↑ Difficulties in weaning from bottle in daughters (delay of 9.6 months) • *Concern about weight of daughters ↑ (age 2–5)	8/9
Allen et al. 2014	Mothers with or without history of EDs and at least one child	*n* = 18 ED (active ED = 8) *n* = 148 NED	11% ED	Prospective cohort study	Australia	Early feeding practices, child ED symptoms and child psychological well-being	EDE-I-12 (current symptoms), parent interview (history of ED)	Parent interview: early feeding practices, duration of BF, age of introduction to solids	Compared to controls*:* • *Women with ED introduced child to solids earlier (15.96 weeks ±1.79 vs. 21.86 ± 0.49 weeks) • No difference in age at weaning (age where BF stopped) (36.09 weeks ±6.25 vs. 39.75 weeks ± 1.66) • No difference in BF practices between daughters and sons	7/9
Brinch et al. 1988	Former AN patients	*n* = 50	100% AN	Follow-up of the Copenhagen Anorexia Nervosa Follow-Up Study	Denmark	Parental functioning of former AN patients (retrospective)	In the past by the hospital they were treated	Semi-structured interview about history of pregnancies, development of children, BF behavior	Compared to Danish average: • Same mean duration of BF • 84% of mothers breastfed for an average of 15 weeks • Reasons for weaning: too little milk, no milk, mastitis, physical reasons, dislike BF	4/9
Evans and Le Grange 1995	Mother-child dyads with or without history of EDs, children > 7 years	*n* = 10 ED (active ED = 4) *n* = 10 NED	50% AN or BN	Retrospective cohort study	South Africa	Effect of maternal ED on feeding style, BF duration and emotions during feeding	Questionnaires: EAT + Body Shape Questionnaire	Semi-structured interview about development of child, eating and BF behavior	Compared to controls: • Similar durations of BF (7.6 vs. 7 months) • Initial BF: 81.25% (ED) vs. 90% (control) • 55.6% scheduled feeding vs. 10% (control) • Emotional problems during BF: feeling unsuccessful, guilt, “waves of sadness” • Problems with BF: insufficient lactation, baby’s allergy to breast milk	7/9
Hoffman et al. 2014	Mother-child dyads with or without history of EDs	*n* = 25 ED *n* = 25 NED	50% AN or BN or OSFED, 50% NED	Non-randomized cohort study	USA	Feeding styles, child diet, restrictive approaches during feeding of mothers with history of EDs	Interview: SCID-I/P Questionnaire: EDE-Q	IFSQ, modified Toddler Diet Questionnaire	Compared to controls*:* *•* No difference in overall duration of BF (11.92 ± 5.69 months (ED) vs. 12.87 ± 5.72 months (control)) *•* No difference in percentage of mothers who initially breastfed • No difference in percentage of mothers who breastfed >1 month • No difference in age of introduction to solid food	7/9
Larsson and Andersson-Ellström 2003	Mother-child dyads with or without history of EDs	*n* = 52 ED *n* = 402 NED	11% ED	Cross-sectional study	Sweden	Progress and experiences of BF and length of BF periods	Self-report questionnaire	Self-report questionnaire of 40 items: BF activity, experiences of BF and of changes of the breasts	Compared to controls: • *Fewer women exclusively breastfed at 3 months (73% (ED) vs. 84% (control)) • No BF at 3 months: ↑ in ED mothers (19%) vs. controls (7%) • Weak tendency: more negative experiences with breast changes and less positive feelings in connection with BF	6/9
Martini et al. 2019	Pregnant women with or without history of EDs	*n* = 25 current ED *n* = 28 past ED *n* = 46 NED	53% ED	Longitudinal study (NEST-p)	UK	Comparison of women with C-ED or P-ED to healthy controls in relation to BF and maternal feeding attitudes at 8 weeks and 6 months postnatally	Interview: SCID-IV-TR Axis 1 Disorder at baseline Questionnaire: EDE-Q (36 items) at every measurement point	IFQ (28 items), primary feeding method at 8 weeks/6 months	C-ED and P-ED compared to controls: • No difference: percentage of mothers exclusively BF at 8 weeks (64.4% HC/66.7% P-ED/52% C-ED) • No difference: percentage of mothers exclusively or partially BF at 6 months (66.7% HC/73.9% P-ED/57.1% C-ED) • P-ED less awareness about infant hunger and satiety; high concern about infant becoming overweight at 8 w/6 m • C-ED high concern about infant becoming overweight at 6 m • Scheduled feeding slightly higher for C-ED at 6 m	7/9
Micali et al. 2009	Mother-child dyads with or without history of EDs and with or without other psychiatric problems	*n* = 12,050 (329 AN/194 BN/10,379 HC/1148 other)	2.7% AN, 1.6% BN	Longitudinal analysis of the ALSPAC study	UK	Feeding difficulties at age 1 and 6 months and BF during first year of life in mothers with history of ED compared to women with or without other psychiatric disorders	Self-report questionnaire about recent or past psychiatric problems	Questionnaire about BF at 1, 6, 15 months postnatally: feeding behaviors and difficulties	AN and BN compared to controls: • More likely to start BF (83% vs. 76%) • *Less likely to stop BF in the first year *•* BN most likely to continue BF, longer BF duration because of infant’s higher refusal to eat solid food • AN more early onset feeding difficulties • Infants of women with ED: similar rate of fast drinking	7/9
Nguyen et al. 2017	Mother-child dyads with or without history of EDs	*n* = 591 ED *n* = 5605 NED	9.5% AN or BN	Longitudinal analysis of the Generation R study	The Netherlands	Association of maternal history of ED with diet quality and BF practices	Self-report questionnaire for AN and BN	Postnatal questionnaire about BF behavior at 2, 6, 12 months	Compared to controls*:* *•* Less likely to initiate BF (not * after adjustment for covariates: maternal age, ethnicity, educational level, BMI, net household income, and psychiatric symptoms) • No difference in duration of BF (among mothers who breastfed)	6/9
Popovic et al. 2018	Women with a history of AN or BN or no ED, at least one child	*n* = 281 ED *n* = 4800 NED	1.9% AN and/or BN (*n* = 100), 5.5% AN and/or BN and/or purging (*n* = 281)	Analysis of the NINFEA birth cohort study	Italy	Association of maternal history of ED with infant wheezing	Self-report questionnaire about lifetime ED diagnosis	Online questionnaires during pregnancy, at 6 + 18 months postpartum: BF more or less than 6 months?	Compared to controls*:* • More likely to BF less than 6 months	6/9
Torgersen et al. 2010	Mother-child dyads with or without history of EDs	*n* = 2422 ED *n* = 36,933 NED	0.1% AN (*n* = 39), 0.8% BN (*n* = 334), 4.6% BED (*n* = 2007), 0.1% EDNOS-P (*n* = 42)	Longitudinal analysis of the MoBa study	Norway	Prevalence of BF between women with any ED subtype and mothers without ED in the first 6 months after birth	Self-report questionnaire after DSM-IV criteria	Questionnaire 6 months after birth: BF practices, bottle feeding, introduction of other drinks and solid food	Compared to controls*:* • 98% initially BF, no difference between groups • No difference in risk of early cessation of exclusive BF • Risk of early cessation before 6 months * increased for AN and EDNOS-P)	6/9
Torgersen et al. 2015	Mother-child dyads with or without history of EDs	*n* = 3013 ED *n* = 50,866 NED	0.1% AN (*n* = 44), 0.9% BN (*n* = 436), 5.0% BED (*n* = 2475), 0.1% EDNOS-P (*n* = 58)	Longitudinal analysis of the MoBa study	Norway	Dietary feeding patterns of women with ED compared to the diet of children of mothers without ED	Self-report questionnaire after DSM-IV criteria	Questionnaire 6 months after birth: BF practices (stopped BF = 0, still BF = 1)	*Compared to controls:* • BF rates 6 months postpartum: AN 58%, BN 79%, BED 76%, EDNOS-P 59%, no-ED 82%	6/9
Waugh and Bulik 1999	Women with or without history of EDs, children between 12 and 48 months, each group 5 boys and 5 girls	n = 10 ED (6 AN, 7 BN) (active ED = 6) *n* = 10 NED	30% AN (*n* = 3), 40% BN (*n* = 4), 30% AN and BN (*n* = 3)	Longitudinal study	New Zealand	Health and development, temperament, body satisfaction, nutritional status of children, mealtime interaction patterns in offspring 1–4 years	Either Diagnostic Interview for Genetic Studies or Structured Clinical Interview for DSM-III-R	Interview: initial feeding method, reason for bottle feeding (+ Child’s Plunket Book)	Compared to controls*:* • Initial BF: 8 ED mothers vs. 10 control • ED mothers more difficulties maintaining BF * BF until weaning: 6 ED mothers vs. 10 control • 2 ED mothers ceased BF within 6 weeks because of BF problems: embarrassment, insufficient milk amounts • 2 ED mothers no BF at all: embarrassment, depression	8/9

Abbreviations: *NOS* Newcastle Ottawa Scale, *ED* eating disorder, *NED* non-eating disorder, *C-ED* current eating disorder, *AN* anorexia nervosa, *BN* bulimia nervosa, *BED* binge eating disorder, *OSFED* other specified feeding and eating disorders, *HC* healthy controls, *ALSPAC* Avon Longitudinal Study of Parents and Children, *MoBa* Norwegian Mother and Child Cohort Study, *NINFEA* Nascita ed INFanzia: gli Effetti dell’Ambiente, *BF* breastfeeding, *EDE-I/Q* Eating Disorder Examination-Interview/Questionnaire, *EAT* Eating Attitudes Test, *DSM* Diagnostic and Statistical Manual of Mental Disorders, *SCID* Structured Clinical Interview for DSM Axis I Disorders; *significant finding (*p* < 0.05); *TFEQ* Three-Factor Eating Questionnaire, *IFR* Infant Feeding Report, *IFSQ* Infant Feeding Styles Questionnaire, *IFQ* Infant Feeding Questionnaire

## Discussion

This systematic review analyzed the BF practices of mothers with EDs. Thirteen publications assessed total duration and initial BF, five of the papers analyzed emotions and/or problems of mothers during BF, and three of the publications investigated the incidence of scheduled feeding among mothers with EDs.

### Breastfeeding initiation and total duration of breastfeeding

In terms of BF initiation and the total duration of BF, the relevant publications found very differing results. Most studies showed significant evidence or at least strong tendencies toward a similar total duration of BF between mothers with and without EDs ( Agras et al. 1999 (*p* > 0.05); *n* = 194; Allen et al. 2014 (*p* > 0.05); *n* = 166; Brinch et al. 1988 (*p* > 0.05); *n* = 50; Evans and Le Grange 1995 (*p* > 0.05); *n* = 20 ; Hoffman et al. 2014 (*p* = 0.67); *n* = 50 ; Martini et al. 2019 (*p* > 0.05); *n* = 99 ; Nguyen et al. 2017 (*p* > 0.05); *n* = 6196 ). The overall sample size for these seven studies was *n* = 788 for mothers with EDs and *n* = 5987 for controls.

On the contrary, five publications with a considerably higher number of participants reported shorter duration of BF for mothers with a history of EDs (Larsson and Andersson-Ellström 2003; *n* = 454; Popovic et al. 2018; *n* = 5081; Torgersen et al. 2010; *n* = 39,355; Torgersen et al. 2015; *n* = 53,879; Waugh and Bulik 1999; *n* = 20). The overall sample of mothers with EDs for these five publications was *n* = 3356 and *n* = 56,078 for controls. The main reasons for women with ED to stop BF were insufficient milk quantities or negative experiences with BF (Waugh and Bulik 1999; *n* = 20). It must be considered that this information was self-reported and reasons for insufficient milk might be of medical or subjective nature. Moreover, the infant’s hunger and satiety cues are subject to the mother’s interpretation and do not necessarily reflect the child’s exact condition of hunger or satiety. Physical reasons for weaning might not necessarily be caused by the ED, because insufficient milk quantities, sore nipples, and other physical symptoms are also common in healthy mothers (Kersting et al. 2020). Psychological aspects seem to be more relevant for the decision to stop BF. Furthermore, the support of family, partner, and/or community, the working situation of the mother, and the values and influences of the social environment and culture can play a big role in BF behavior in general (Rempel and Rempel 2004).

Only one large prospective population-based study found a longer BF duration in mothers with EDs, more specifically in mothers with BN (Micali et al. 2009; *n* = 12,050). Especially for mothers with BN, BF might prevent from dysfunctional eating behaviors (Stapleton et al. 2008; *n* = 16).

Studies that investigated BF initiation in mothers with EDs included studies with very small sample sizes (Evans and Le Grange 1995; *n* = 20; Hoffman et al. 2014; *n* = 50; Waugh and Bulik 1999; *n* = 20). Three large population-based studies led to three different results: Torgersen et al. (2010) found a similar rate of initial BF in both groups (Torgersen et al. 2010; *n* = 39,355), Micali et al. (2009) found that mothers with EDs were significantly more likely to start BF (Micali et al. 2009; *n* = 12,050), and Nguyen et al. (2017) stated that mothers with EDs were less likely to start BF (not significant) (Nguyen et al. 2017; *n* = 6196). Reasons for these conflicting results may be the difference in ED samples between studies and different methodological approaches to determine the diagnosis of EDs and BF practices.

### Emotions during breastfeeding and general attitudes toward breastfeeding

Summarizing the findings of three relevant publications, reported emotions during BF were often negative or dominated by concerns (Evans and Le Grange 1995; *n* = 20; Larsson and Andersson-Ellström 2003; *n* = 454; Waugh and Bulik 1999; *n* = 20). It should again be noted that the results have limited power due to rather small sample sizes. As this is an emotionally loaded and very subjective topic, a retrospective method of assessment is disadvantageous (Evans and Le Grange 1995; *n* = 20). Answers could be adapted to social desirability or underlie recall biases, especially if the child is already beyond early childhood (Evans and Le Grange 1995; *n* = 20). A qualitative approach would be appropriate to give real-life impressions of the emotions felt during BF of mothers suffering from EDs (Stapleton et al. 2008, 2009). Since BF is related to food and nutrition, it is not surprising that besides the maternal pride to be able to feed the child, there is also fear and insecurity in mothers with EDs. A negative self-image and lacking trust in their own body (Stice and Shaw 2002) are also possible reasons for negative feelings during BF. In addition, some mothers with EDs started to worry very early about the weight of their newborn daughters (Agras et al. 1999; *n* = 194), which also contributes to general feelings of concern during the time of BF.

### Problems associated with breastfeeding

All of the five relevant studies identified problems with BF in mothers with EDs (Agras et al. 1999; *n* = 194; Brinch et al. 1988; *n* = 50; Evans and Le Grange 1995; *n* = 20; Micali et al. 2009; *n* = 12,050; Waugh and Bulik 1999; *n* = 20). Problems with BF are generally common and do also occur frequently in healthy women (Gianni et al. 2019; Kent et al. 2015). Micali et al. (2009) were able to show differences between AN and BN for problems regarding BF. Children of mothers with AN showed more overall difficulties and children of mothers with BN refused solids, which led to longer BF duration (Micali et al. 2009; *n* = 12,050). This contrasts the findings of Stapleton et al. (2008) that cycles of bingeing and purging in BN patients led to early cessation of BF in some women (Stapleton et al. 2008; *n* = 16). However, one must consider that the ED sample differed between the two studies regarding ED severity. Agras et al. (1999) evaluated daughters and sons separately, with the result that daughters showed significantly more vomiting and fast sucking (Agras et al. 1999; *n* = 194). It should be noted that these observations were made under laboratory feeding conditions, which might not represent the real feeding situation at home.

In any case, it may be stated that there is a tendency toward more problems associated with BF for mothers with any subtype of ED, which represents a burden for the mother and the child. As we have already pointed out in “Breastfeeding initiation and total duration of breastfeeding”, physical problems associated with BF do not necessarily seem to be related to an ED, but are generally common (Kersting et al. 2020). Emotional problems have a great magnitude in mothers with EDs (see “Emotions during breastfeeding and general attitudes toward breastfeeding”). These negative feelings might also be perceived by the child, which then expresses its discomfort by altered behavior.

Recently, it has been outlined that oxytocin also contributes to the regulation of eating behavior and might play a role in the disease process of EDs. Oxytocin functioning might partly underlie BF difficulties seen in women with EDs and might even contribute to an intergenerational transmission of EDs via attachment experiences in early childhood (Giel et al. 2018). In this context, the type of delivery should be considered in studies, since cesarean sections alter the oxytocin levels after birth, which can affect the mother’s BF behavior (Marchini et al. 1988).

### Scheduled feeding

Concerning the incidence of scheduled feeding, all three relevant studies showed different results while analyzing slightly different subgroups (Agras et al. 1999; *n* = 194; Evans and Le Grange 1995; *n* = 20; Martini et al. 2019; *n* = 99). Unfortunately, there was no definition of scheduled feeding. Only Evans and Le Grange (1995) gave information about the source of the schedules. This was also the only study that found a significantly higher implementation of scheduled feeding in mothers with EDs (Evans and Le Grange 1995; *n* = 20). Further research is urgently needed to be able to draw a conclusion on this issue, especially with distinction between the subtypes of EDs, because the different characteristics of ED subtypes might lead to contrasting results in terms of scheduled feeding. The question arises whether scheduled feeding is disadvantageous or beneficial for the child. Regulated feeding times could possibly be helpful for mothers with EDs to establish a steady rhythm and to improve recognition of the infant’s hunger and satiety signals (Evans and Le Grange 1995; *n* = 20). Furthermore, there might be cases where feeding on demand is detrimental for the mother’s health, for example at night. In this case, regulated feeding times could be beneficial for the mother’s sleep and regeneration. BF in general can also be incompatible with other needs of the mother, for instance if the mother wishes to return to work early after birth. These are examples where planned feeding and/or replacing BF with formula can unburden the mother, since she can give this task to her partner or someone else. However, the current recommendation on breastfeeding frequency is to breastfeed on demand (Koletzko et al. 2010).

### Strengths and limitations of the included studies

Five of the publications were based on large cohort and/or publication-based analyses (Micali et al. 2009; *n* = 12,050; Nguyen et al. 2017; *n* = 6196; Popovic et al. 2018; *n* = 5081; Torgersen et al. 2010; *n* = 39,355; Torgersen et al. 2015; *n* = 53,879); most of the other studies had very small sample sizes, and therefore limited generalizability. All publications included a control group with mothers without EDs. Mothers with EDs were partly overrepresented in comparison to the overall population, which might be caused by a special interest of affected mothers in this topic (Agras et al. 1999; *n* = 194). The major strength of the studies with large sample sizes beside the study population number was prospectivity and therefore a minimization of recall bias. Furthermore, these studies partly applied DSM-V criteria for ED diagnosis and included numerous covariates in the analysis (Nguyen et al. 2017; Torgersen et al. 2010; Torgersen et al. 2015). Two of the studies had representative samples in terms of the incidence of ED subtypes (Micali et al. 2009; Popovic et al. 2018). Limitations included self-report ED diagnoses in all the studies (Micali et al. 2009; Nguyen et al. 2017; Popovic et al. 2018; Torgersen et al. 2010; Torgersen et al. 2015) and inclusion of only two ED subtypes (AN/BN) in two studies (Micali et al. 2009; Popovic et al. 2018).

During the long period of time that the publications cover, the public attitude toward BF might have changed. This could be one reason for shorter overall BF durations in the 1980s in both groups of women compared to recent years. Publication dates ranged from 1988 (Brinch et al. 1988; *n* = 50) to 2019 (Martini et al. 2019; *n* = 99), which also resulted in different versions of diagnostic criteria for EDs being applied (). In four cases, no DSM criteria were applied (Brinch et al. 1988; *n* = 50; Larsson and Andersson-Ellström 2003; *n* = 454; Micali et al. 2009; *n* = 12,050; Popovic et al. 2018; *n* = 5081). The ED diagnosis was based on self-report in most of the studies which might cause a sample bias, because women who reported EDs might have milder forms and severe cases might not be reported due to feelings of shame. Not all studies differentiated active, lifetime, or subtypes of EDs. Only Martini et al. (2019) differentiated results for current ED and past ED (Martini et al. 2019; *n* = 99), and only Torgersen et al. (2010, 2015) and Micali et al. (2009) differentiated between subtypes of EDs (Micali et al. 2009; *n* = 12,050; Torgersen et al. 2010; *n* = 39,355; Torgersen et al. 2015; *n* = 53,879).

The measurements on BF were conducted at very different time points, some at 8 weeks and 6 months postpartum (Martini et al. 2019; *n* = 99), others 3 days per month until the child was weaned (Agras et al., 1999; *n* = 194), and others collected retrospective information on BF without a specific time point (Brinch et al. 1988; *n* = 50; Evans and Le Grange 1995; *n* = 20; Waugh and Bulik 1999; *n* = 20). Retrospectivity in general involves the risk of recall bias and adaptation, especially when it comes to such sensitive topics like EDs and BF (Brinch et al. 1988; *n* = 50). A limitation of the studies investigating BF durations was the missing differentiation between exclusive and partial BF (Thulier 2010). Only Torgersen et al. (2010, 2015) named the type of BF which was measured (Torgersen et al. 2010; *n* = 39,355; Torgersen et al. 2015; *n* = 53,879). This leads to difficulties in the interpretation and comparability of total durations of BF. It can be suspected that results with durations of 3 months or less are meant for exclusive BF, but this cannot be validated.

EDs are often accompanied by other psychological diseases like perinatal mood and anxiety disorders (Makino et al. 2020). These could influence the decision to initiate, continue, or stop BF (Vieira et al. 2018) and should therefore be carefully considered when examining BF mothers with EDs in future studies. Also, the type of treatment should be indicated, for example, medication or psychotherapy. A prolonged stay in the hospital after birth, lacking social support, and type of delivery are factors that might contribute to the mother’s decision to BF or not (Paiva et al. 2013; Sayres and Visentin 2018). Also, the duration of BF could be influenced by several social covariates (Kelly et al. 2006). The included studies in this review hardly considered these numerous factors when analyzing the BF behavior of women with and without EDs.

### Strengths and limitations of the present review

This review is the first to examine BF practices in mothers with EDs in such detail. The strengths of this review are its methodical and systematic approach. This review is one of very few systematic reviews on BF in combination with maternal EDs (Behar and Arancibia 2014; Martini et al. 2020). Both Behar and Arancibia (2014) and Martini et al. (2020) had a different research focus by covering BF in mothers with EDs as one of many topics, and therefore, the number of included studies on this topic was limited in both reviews. In this present review, the specific focus is solely on BF practices in mothers with and without EDs, with a systematic approach and high actuality. We defined a relatively broad search term in order to be as inclusive as possible and to cover a large body of research on this topic. In addition to the duration of BF, problems and emotional status of the affected mothers were also analyzed in this review. Due to the large heterogeneity of the included studies and publications, it was not possible to perform a meta-analysis.

## Conclusion and further directions

Based on the research questions for the present systematic review, the main conclusions are as follows: (a) the studies included in this review showed mixed results on BF duration, but due to the results of larger studies, it is reasonable to assume that women with EDs (especially AN) breastfeed for a shorter total duration as healthy women. There is not enough evidence to evaluate initial BF in women with EDs. (b) Women with EDs showed a higher rate of problems during BF compared to healthy women. Since physical problems are also common in healthy mothers, emotional problems are an especially relevant factor in mothers with EDs.

The results of the included studies are very heterogeneous, and methods are hardly comparable. Therefore, it was not possible to calculate average BF durations for all participants. In addition, a differentiation between exclusive and partial BF was missing, which makes it impossible to rate whether the recommendations for exclusive BF have been achieved.

A drawback of all cited studies is the neglected inclusion of the women’s partners. Studies show that a pregnant woman’s decision on BF is strongly influenced by the attitude of her partner (Rempel and Rempel 2004). Therefore, further studies should assess the attitudes toward BF of paternal or maternal figures and if they support the women’s BF plans. Especially in the case of women with EDs, the partners might be a protective factor for normalizing infant nutrition while setting healthy nutritional norms and being a positive role-model (Keel et al. 1997; Sadeh-Sharvit et al. 2019).

BF is a complex procedure, which does require not only knowledge and support but also confidence in the body and in the body’s capacity to nurture a child (Arora et al. 2000). This confidence is often missing in women suffering from EDs (Stice and Shaw 2002). Women with AN might also doubt that they can give any milk because of their low BMI. However, there is evidence that the amount of breast milk is independent from the maternal BMI ( Prentice et al. 1994).

Feeding the infant is much more than just providing caloric intake; it is one of the most important forms of communication between mother and child and a basis for attachment (Silva et al. 2016). Shorter overall BF and BF problems in mothers with EDs might therefore occur on a continuum of complex difficulties which develop around mother-infant-interactions and reach beyond the mere feeding situation. Therefore, obstetricians, midwives, and other healthcare professionals should begin teaching about breastfeeding prenatally while involving the partners of the women from the beginning. Pregnant women with EDs might be ambivalent toward BF (Stapleton et al. 2008; Stapleton et al. 2009) and motherhood (Koubaa et al. 2008). Furthermore, there is evidence that early negative BF experiences increase the risk for depressive symptoms 2 months postpartum (Watkins et al. 2011). Moreover, many women experience ED relapses and even an increase in severity of their ED symptoms in the postpartum period (Makino et al. 2020). It might also be helpful to seek psychological counseling at the end of the third trimester to counteract or prepare for a possible worsening of ED symptoms. Therefore, monitoring the women’s mental health, their ED pathology, and their adjustment to motherhood should be carried out regularly by providers during this high-risk period (Harris 2010). Women should be reassured that all women are affected by postpartum feelings and emotions when they become mothers which can normalize postpartum experiences and reduce anxiety in affected women (Chizawsky and Newton 2006). Lastly, the involvement of a pediatrician to carefully monitor the nutritional status of the infant should also be considered (Harris 2010).

On the other side, it is important to note that there is a small percentage of mothers of 5 to 10% who are physically not able to breastfeed, although they want to (Kersting et al. 2020). In addition, some women face barriers to BF including demographic, social, economic, and cultural factors (Kelly et al. 2006). No matter why mothers are not able to breastfeed or decide not to breastfeed, they suffer from increasing social pressure and desirability to breastfeed their infants (Diez-Sampedro et al. 2019; Stapleton et al. 2008).

To conclude, it should be considered that not only women who suffer from severe EDs may have problems with or negative feelings toward BF but also women who are dissatisfied with their weight or shape (Barnes et al. 1997; Foster et al. 1996). Professional pre- and postnatal care should therefore address every woman, in the best case including their partners, as part of the routine prenatal care.

## Appendix

Search term (PubMed).

((“breast feeding”[MeSH Terms] OR (“breast”[All Fields] AND “feeding”[All Fields]) OR “breast feeding”[All Fields] OR “breastfeeding”[All Fields]) OR (“lactation”[MeSH Terms] OR “lactation”[All Fields] OR “breast feeding”[MeSH Terms] OR (“breast”[All Fields] AND “feeding”[All Fields]) OR “breast feeding”[All Fields]) OR ((“infant, new-born”[MeSH Terms] OR (“infant”[All Fields] AND “newborn”[All Fields]) OR “new-born infant”[All Fields] OR “neonate”[All Fields]) AND feeding[All Fields]) OR ((“in-fant, newborn”[MeSH Terms] OR (“infant”[All Fields] AND “newborn”[All Fields]) OR “newborn infant”[All Fields] OR “newborn”[All Fields]) AND feeding[All Fields]) OR ((“infant, newborn”[MeSH Terms] OR (“infant”[All Fields] AND “newborn”[All Fields]) OR “newborn infant”[All Fields] OR “baby”[All Fields] OR “infant”[MeSH Terms] OR “infant”[All Fields]) AND feeding[All Fields]) OR ((“infant”[MeSH Terms] OR “infant”[All Fields]) AND feeding[All Fields])) AND ((“feeding and eating disorders”[MeSH Terms] OR (“feeding”[All Fields] AND “eating”[All Fields] AND “disorders”[All Fields]) OR “feeding and eating disorders”[All Fields] OR (“eating”[All Fields] AND “disorders”[All Fields]) OR “eating disorders”[All Fields]) OR (“anorexia nervosa”[MeSH Terms] OR (“anorexia”[All Fields] AND “nervosa”[All Fields]) OR “anorexia nervosa”[All Fields]) OR (“bulimia nervosa”[MeSH Terms] OR (“bulimia”[All Fields] AND “nervosa”[All Fields]) OR “bulimia nervosa”[All Fields]) OR (“binge-eating disorder”[MeSH Terms] OR (“binge-eating”[All Fields] AND “disorder”[All Fields]) OR “binge-eating disorder”[All Fields] OR (“binge”[All Fields] AND “eating”[All Fields] AND “disorder”[All Fields]) OR “binge eating disorder”[All Fields]) OR osfed[All Fields]).
